# *TP53* mutations and number of alterations correlate with maximum standardized uptake value (SUVmax) determined by positron emission tomography/computed tomography (PET/CT) [^18^F] fluorodeoxyglucose (^18^F-FDG PET)

**DOI:** 10.18632/oncotarget.24508

**Published:** 2018-02-16

**Authors:** Geraldine H. Chang, Razelle Kurzrock, Lisa Tran, Maria Schwaederle, Carl K. Hoh

**Affiliations:** ^1^ Department of Radiology, Nuclear Medicine UC San Diego Health, San Diego, California, USA; ^2^ Center for Personalized Cancer Therapy, UCSD Moores Cancer Center, La Jolla, California, USA

**Keywords:** TP53, personalized cancer therapy, PET/CT, SUVmax

## Abstract

**Background:**

Our study explored the relationship between the molecular changes in cancer and the maximum standardized uptake value (SUVmax) determined by positron emission tomography/computed tomography (PET/CT) with [^18^F] fluorodeoxyglucose (^18^F-FDG).

**Results:**

A higher SUVmax correlated with *TP53* alterations, but not with histologic diagnosis or other gene/pathway mutations or copy number alterations. In data from breast, lung and colon cancer, patients with the highest SUVmax show more genomic anomalies compared to those with the lowest SUVmax (*P* < 0.005).

**Conclusions:**

A higher SUVmax on ^18^F-FDG PET/CT is associated with TP53 tumor suppressor gene anomalies and the presence of more genomic anomalies. Since TP53 alterations and high SUVmax both correlate with a poor prognosis, the underlying mechanism/implications of this association merit further study.

**Methods:**

Overall, 176 patients with diverse cancers had a tumor biopsy within 6 months after a PET/CT image for SUVmax measurement. The biopsy was interrogated by next generation sequencing (182 to 315 genes). *TP53, EGFR, ALK, MYC, MET* and *FGF/FGFR* genes and DNA repair, PI3K/Akt/mTOR (PAM), MEK, CYCLIN, and WNT pathway genes were analyzed.

## INTRODUCTION

Cancer is the second most common cause of death in the US, exceeded only by heart disease [1]. Current trends demonstrate a decline in four of the most common cancer types—lung, colorectal, breast and prostate [1–3]. Such improvements reflect both the earlier diagnosis of certain cancers and improvements in treatments.

One area of intense research is the use of comprehensive genomic profiling with the use of next generation sequencing [4–5]. The goal is to use this information to expand treatment options by matching an individual patient with targeted therapies and clinical trials that are relevant to the molecular changes in the tumor [6–7].

In our study, we evaluated our database of patients who have undergone testing for somatic genomic alterations and correlated the results with PET/CT imaging, which was performed using ^18^F-FDG PET/CT. The SUVmax is the most common semi-quantitative parameter determined by ^18^F-FDG PET/CT, and a decrease of SUVmax is associated with a positive response to treatment. ^18^F-FDG PET/CT is a common non-invasive pharmacodynamic marker to assess response to a wide array of anticancer agents among multiple different types of malignancies. We explored, to our knowledge for the first time, the relationship between diverse molecular changes and SUVmax across different malignancies.

## RESULTS

### Patients

We studied 176 patients (109 women, 67 men) aged 23 to 85 years (mean age = 57 years) with the diagnosis of: breast cancer 41 (23%), lung cancer 44 (25%); gastrointestinal cancer 39 (22%), and a variety of other tumor types. Due to the retrospective nature of this study, the patients are in different stages of their cancers and the lesion biopsied included primary and metastatic lesions.

### SUVmax as a linear variable

SUVmax as a linear variable was analyzed using a univariable test—the Mann-Whitney test. The overall mean for SUVmax was 8.30 ± 7.65. The overall median for SUVmax in the *N* = 176 patients = 6.4 (5.8–7.7). There was no significant difference in the SUVmax in the patients when grouped by their diagnosis: breast cancer 7.23 ± 4.12, lung cancer 7.97 + 5.09, and GI cancers 7.40 ± 4.54.

### Genomic anomalies (Table 1)

**Table 1:** Absence or presence of DNA alterations and SUVmax, *N* = 176 patients

Gene^*^	no alteration SUVmax (CI)	with alteration SUVmax (CI)	*P* (2-tailed)
***TP53***	*N* = 92 5.85 (5.0–6.6)	*N* = 84 7.85 (6.1–9.95)	0.023
***DNA Repair***	*N* = 164 6.15 (5.7–8.27)	*N* = 12 9.65 (6.55–11.50)	0.132
***EGFR***	*N* = 140 6.4 (5.7–7.6)	*N* = 36 6.5 (5.5–10.5)	0.617
***PAM***	*N* = 118 6.05 (5.45–6.95)	*N* = 58 7.75 (5.8–10.7)	0.062
***MEK***	*N* = 146 6.2 (5.55–7.35)	*N* = 30 7.75 (5.8–10.2)	0.291
***CYCLIN***	*N* = 110 6.55 (5.8–7.9)	*N* = 66 6.15 (5.35–9.15)	0.483
***WNT***	*N* = 151 6.5 (5.8–7.8)	*N* = 25 6.0 (5.1–8.4)	0.842
***MYC***	*N* = 151 6.2 (5.7–7.3)	*N* = 25 7.9 (5.5–9.5)	0.431
***FGF/FGFR***	*N* = 148 6.8 (6.0–7.9)	*N* = 28 5.35 (4.4–8.65)	0.380

^*^Only variables with ≥ 10 patients included.

Abbreviations: PAM = PI3K/Akt/mTOR pathway.

#### *TP53* Gene

Eighty-four of the 176 patients (48%) had alterations in *TP53* and a significantly higher median SUVmax of 7.85 compare to unaltered *TP53* with a reference median SUVmax of 5.85 (*P* = 0.023).

#### DNA repair genes (*BRCA, BRIP, ATM, MMR, MSH, MLH*)

Twelve of 176 patients (7%) had alterations in DNA repair genes. These 12 patients demonstrated a median SUVmax of 9.65 compared to the median SUVmax of 6.15 in the 164 patients with other gene alterations (*P* = 0.130) (but the affected number of patients is small).

#### *EGFR* gene

Thirty-six of 176 patients (20%) had *EGFR* gene anomalies. The median SUVmax of these 36 patient was 6.5 which was not significantly different than those without EFGR gene anomalies (*P* = 1.0).

#### *PI3K/AKT/MTOR* (PAM) genes (*PTEN, PIK3CA, AKT, TSC, CCNB1, MTOR, FBXW2, NF2*)

Fifty-eight of the 176 patients (33%) had abnormalities in PAM pathway genes. The median SUVmax for these 58 patients was 7.75 compared to 6.05 in the patients without abnormal PAM pathway genes. This was not statistically significant (*P* = 0.062).

#### MEK genes (*RAS, RAF, MAPK, CNAS*)

Thirty of the 176 patients (17%) had MEK gene anomalies. Their median SUVmax was 7.75 as compared to 6.20 without the MEK gene anomaly. This was also not significantly different (*P* = 0.291).

#### CYCLIN genes (*CCND, CDK, CDKN, RB*)

Sixty-six of the 176 patients (38%) had abnormalities in CYCLIN pathway genes and a median SUVmax of 6.15 compared to 6.55 in the patient without a CYCLIN gene abnormality. These means were not significantly different (*P* = 0.483).

#### WNT genes (*APC, CTNNB, NOTCH*)

Twenty-five of the 176 patients (14%) had abnormalities in WNT pathway genes and a corresponding median SUVmax of 6.0 compared to a median SUVmax of 6.5 in patients without WNT pathway abnormalities. These median SUVmaxes were not statistically significant, (*P* = 0.842).

#### *MYC* gene

Twenty-five of the 176 patients (14%) had *MYC* gene anomalies and a corresponding median SUVmax of 7.9 as compared to the 151 patients without MYC gene anomalies with an median SUVmax of 6.2. This was not significantly different (*P* = 0.431).

#### *FGF/FGFR* genes

Twenty-eight of the 176 patients (16%) had *FGF/FGFR* gene anomalies and a median SUVmax of 5.35 as compared to the 148 patient without anomalies and an median SUVmax of 6.8. This was not significant (*P* = 0.380).

#### *ALK* and *MET* gene

Three of the 176 patients (2%) had ALK gene anomalies. Four of the 176 patients (2%) had MET gene anomalies. Only variables with ≥10 patients were included and, thus, these gene alterations were not assessed in the univariable analysis.

#### Number of alterations

The average of the three highest and three lowest SUVmaxes and the presence of the various genomic anomalies in the three patient groups—breast cancer, lung cancer, and colon cancer are shown in Table 2. In the three cancers, the three patients with the highest SUVmax show more genomic anomalies compared to the three patients with the lowest SUVmax (*P* < 0.005).

**Table 2:** DNA alterations present in patients with the three highest and three lowest SUVmax lesions in breast cancer, lung cancer, and colon cancer groups

	Breast CA, 3 highest	Breast CA, 3 lowest	Lung CA, 3 highest	Lung CA, 3 lowest	Colon CA, 3 highest	Colon CA, 3 lowest
**Mean SUVmax**	16.1	1.7	16.7	1.7	16.5	2.2
***TP53***	3	1	3	1	3	1
***DNA Repair***						
***EGFR***	2		1	2		
***PAM***	2	1	3		1	
***MEK***					3	
***CYCLIN***	1	2	2		1	
***WNT***		1	2	1		
***MYC***	1	1				
***FGF/FGFR***	2	3				

## DISCUSSION

Oncologic therapies are evolving with increasing emphasis on the evaluation of multiple genomic alterations within the biological pathways driving tumorgenesis, with the idea of providing molecularly targeted therapy. ^18^F-FDG PET/CT is widely accepted as a standard of care for tumor staging/restaging as well as the modality of choice to detect malignancy and predict prognosis. In addition, ^18^F-FDG PET /CT often determines and guides therapeutic decisions and also serves as a way to monitor response to therapy.

The purpose of our study was to determine whether or not ^18^F-FDG PET/CT SUVmax correlated with specific genomic alterations. We therefore compared the SUVmax and the presence of common DNA alterations across different types of malignancies. The genes include *TP53*, DNA repair genes, EGFR, *ALK*, *MYC*, *MET* and *FGF/FGFR* genes as well as key genes in the PAM, MEK, CYCLIN and WNT pathways. We found that only *TP53* gene abnormalities correlated with SUVmax. However, the number of alterations also correlated with SUVmax (Higher number of alterations corresponded with higher SUVmax).

The *TP53* gene provides instructions for making a protein called tumor protein p53, which is a tumor suppressor [8–11]. The p53 protein regulates cell division by preventing cells from growing and dividing too fast. The p53 protein is located in the nucleus of cells where it binds directly to DNA [6–9]. When DNA in a cell becomes damaged, p53 plays a critical role in determining whether the DNA will be repaired or the damaged cell will undergo apoptosis [8–11]. If the DNA can be repaired, p53 activates other genes to fix the damage [8–11]. If the DNA cannot be repaired, p53 prevents the cells from dividing and signals it to undergo apoptosis [8–11]. By stopping cells with mutated or damaged DNA from dividing, p53 helps prevent the development of tumors [8–11].

Studies with *in vitro* cancer cells have shown that mutations in *TP53* are found at high frequencies in several cancer types, including both breast and lung cancers. High uptake of ^18^F-FDG detected using ^18^F-FDG PET has been associated with a poor prognosis. Studies at a cellular level suggest that mutations in *TP5*3 are associated with specific changes in glucose metabolism detected by PET. For instance, a study by Smith *et al.* demonstrated that *in vitro* transfection of breast cancer cells with a dominant negative *TP53* gene construct that reduces the expression of p53 is associated with enhanced ^18^F-FDG uptake [12]. Another report by Brito *et al*. demonstrated that the non-functional expression of the P53 protein leads to higher levels of ^18^F-FDG uptake in a hepatocellular cancer cell line [13]. Of interest, *TP53* gene alterations may be a poor prognostic factor similar to the presence of high SUVmax; as an example, Van der Veldt and colleagues demonstrated that p53 was a biomarker strongly associated with recurrence in cervical cancer [14].

Although there have been multiple studies at a molecular level with malignant cell lines, our study is one of the first and the most extensive study to explore the correlation between molecular markers in tissue biopsies from patients and SUVmax. FDG uptake did not correlate with tumor diagnosis. Nor was any relation found between FDG uptake and the presence of DNA repair genes, *EGFR, MYC, or FGF/FGFR* genes or genes in the PAM, CYCLIN, and WNT pathways. However, the number of patients was small and it is plausible that these limited numbers precluded finding statistical significance.

## CONCLUSIONS

A higher SUVmax on ^18^F-FDG PET was associated with oncogenic alterations in the *TP53* tumor suppressor gene. Abnormalities in *TP53* and increased SUVmax are each considered poor prognostic factors in patients with cancer. A higher SUVmax also correlated with the presence of more oncogenic alterations. Further investigation of the mechanism underlying correlations between genomic anomalies and PET imaging results is warranted.

## MATERIALS AND METHODS

### Patients

We studied 176 consecutive patients with available data who underwent ^18^F-FDG PET/CT six months or less before a biopsy for genomic profiling. The SUVmax of the biopsied lesion was obtained from the PET/CT imaging. Molecular testing data was analyzed to determine the presence of common DNA alterations. For our study, we included *TP53,* DNA repair genes [BRCA, BRIP, ATM MMR, MSH, MLH], *EGFR*, PI3K/Akt/mTOR (PAM) pathway genes [PTEN, PIK3CA, AKT, TSC, CCNB1 MTOR, FBXW2], *CYCLIN* [CCND, CDK, CDKN, RB], WNT pathway [APC, CTNNB, NOTCH], *ALK, MYC*, and FGF/FGFR genes. This study was performed and patients consented in accordance with the guidelines of the UCSD Internal Review Board (PREDICT [Profile Related Evidence Determining Individualized Cancer Therapy], protocol; NCT02478931)

### ^18^F-FDG PET/CT

A combined ^18^F-FDG PET/ CT scanner (General Electric Discovery VCT PET/CT, Waukesha, WI) was used to perform whole body imaging. Images were interpreted according to standard methods. Whole-body CT covers a region ranging from the head to the mid-thigh. After fasting for at least four hours, patients received an intravenous injection of ^18^F-FDG [10–20 mCi]. Blood glucose was checked in all patients before performing ^18^F-FDG PET/CT and no patient had a blood glucose level >160 mg/dl. About 50 minutes later, CT scanning was conducted and whole-body emission PET scanning was performed. Attenuation-corrected PET images were reconstructed with an iterative reconstruction algorithm. ^18^F-FDG PET/CT images were generated for review on a workstation.

### Qualitative analysis of ^18^F-FDG PET /CT

Quantitative analysis was performed on the institution's pictures archiving and communication system (PACS), (AGFA Impax 6.3, Mortsel Belgium). The PACS software was used to draw a single region of interest (ROI) in the area of the lesion with most intense uptake to determine the SUVmax. The SUVmax was determined according to the standard formula, with activity in the region of interest (ROI) being calculated as mCi/ml divided by the injected dose and normalized to the patient's body weight. The SUVmax was defined as the maximum activity within the ROI.

### Genomic analysis

Genomic analysis was performed using a clinical next generation sequencing (NGS) based assay (182 to 315 genes) (FoundationOne™, Foundation Medicine Inc., Cambridge, MA), which includes detection of base substitutions, insertions, deletions, copy number alterations, and selected gene fusions. We included EGFR, PI3K/Akt/mTOR (PAM) pathway genes, CYCLIN, WNT pathway, ALK, MYC, and FGF/FGFR genes.
